# Emergence of Small Colony Variants Is an Adaptive Strategy Used by Pseudomonas aeruginosa to Mitigate the Effects of Redox Imbalance

**DOI:** 10.1128/msphere.00057-23

**Published:** 2023-02-28

**Authors:** Alison Besse, Marie-Christine Groleau, Eric Déziel

**Affiliations:** a Centre Armand-Frappier Santé Biotechnologie, Institut National de la Recherche Scientifique (INRS), Laval, Québec, Canada; Martin Luther University of Halle-Wittenberg Institute of Biology/Microbiology

**Keywords:** RSCV, adaptation, biofilms, cellular redox status, electron acceptor, phenotypic variation

## Abstract

The ability to generate a subpopulation of small colony variants (SCVs) is a conserved feature of Pseudomonas aeruginosa and could represent a key adaptive strategy to colonize and persist in multiple niches. However, very little is known about the role of the SCV phenotype, the conditions that promote its emergence, and its possible involvement in an adaptive strategy. In the present work, we investigated the *in vitro* selective conditions promoting the emergence of SCVs from the prototypical strain PA14, which readily forms SCVs in nonagitated standing cultures. We found that O_2_ limitation, which causes a redox imbalance, is the main factor selecting for the SCV phenotype, which promotes survival of the population *via* formation of a biofilm at the air-liquid interface to access the electron acceptor. When this selective pressure is relieved by aeration or supplementation of an alternative electron acceptor, SCVs are barely detectable. We also observed that SCV emergence contributes to redox rebalancing, suggesting that it is involved in an adaptive strategy. We conclude that selection for the SCV phenotype is an adaptive solution adopted by P. aeruginosa to access poorly available O_2_.

**IMPORTANCE** The bacterium Pseudomonas aeruginosa is an opportunistic pathogen that thrives in many environments. It poses a significant health concern, notably because it is a causative agent of nosocomial infections and the most prevalent pathogen found in the lungs of people with cystic fibrosis. In infected hosts, its persistence is often related to the emergence of an alternative phenotype known as small colony variant (SCV). Identification of conditions selecting for the SCV phenotype contributes to knowledge regarding adaptive mechanisms exploited by P. aeruginosa to survive in multiple niches and persist during infections. Hindering this adaptation strategy could help control persistent P. aeruginosa infections.

## INTRODUCTION

The bacterium Pseudomonas aeruginosa thrives in diverse environments, such as aquatic habitats, soil, food, and even anthropogenically constructed environments, such as hospital premise plumbing systems (1–5). In addition to environmental habitats, P. aeruginosa strains are frequently isolated from clinical samples. It is one of the most frequent causative agents of nosocomial infections and a major cause of infections among immunocompromised individuals, especially in people with cystic fibrosis (CF) (6–8). P. aeruginosa is also capable of infecting nonmammalian hosts, such as plants and insects (9–11). To persist in these various environments, P. aeruginosa has to face hostile conditions such as poor nutrient availability, O_2_ limitation, and desiccation, along with host immunity and antimicrobial treatments in clinical contexts.

The successful adaptation of P. aeruginosa to multiple niches is attributed to its high genomic and metabolic versatility (12). For instance, the flexible metabolism of P. aeruginosa allows it to use different final electron acceptors to produce energy usable by the bacteria. Although energy generation is based mainly on oxidative substrate catabolism, P. aeruginosa is able to grow under anaerobic conditions with alternative electron acceptors, such as nitrate, or to survive by fermentation of arginine or pyruvate (13–15). Activation of the denitrification pathway is regulated by Anr, a direct O_2_ sensor that regulates anaerobic gene expression (16). When P. aeruginosa undergoes electron acceptor limitations during *in vitro* biofilm growth, it develops different strategies to mitigate low O_2_ conditions. These strategies include the production of small redox molecules, called phenazines, which act as carriers shuttling electrons from intracellular metabolism to distant extracellular oxidants (17, 18). Phenazines behave as alternative electron acceptors, and their production facilitate intracellular redox balancing by oxidizing redox state (19). In the absence of phenazines, P. aeruginosa can still cope with O_2_ limitation by changing the overall structure of a biofilm to increase the surface exposed to O_2_ (20, 21); this is visible as wrinkles on the surface of colony biofilms (20).

However, when P. aeruginosa undergoes electron acceptor limitations during *in vitro* liquid standing cultures (22), adaptive mechanisms enabling its growth and survival to mitigate O_2_ limitation are poorly understood. When grown in standing conditions, P. aeruginosa adopts an alternative phenotype called small colony variants (SCVs). SCVs are characterized by the smaller size of their colony compared to wild-type (WT) colonies. Beside their size, SCVs display several specific properties that distinguish them from WT colonies. SCVs exhibit cell surface hyperpiliation and stronger adherence to abiotic surfaces (23–25). These properties promote biofilm formation (22, 26). Consistent with enhanced biofilm formation, an overproduction of exopolysaccharides (EPS) (Pel and/or Psl), the major component of the biofilm matrix, as well as c-di-GMP, has also been reported in SCVs (22, 27). Several studies have reported stable genetic mutations in the c-di-GMP signaling pathway in SCV genomes, which led to the switch to SCV phenotype (22, 28–30). The phenotypic reversion of SCVs to a parental-like phenotype, occurring *in vitro* or *in vivo*, is due to a second loss-of-function mutation (22, 31). Although frequently associated with persistent P. aeruginosa infections in individuals with CF (21, 32), SCVs are not restricted to a clinical context. Strains isolated from various nonclinical environments such as soil, food, and hospital water systems can also form SCVs (22, 23). Since the ability to adopt a SCV phenotype is widely distributed among P. aeruginosa strains, this could represent a key adaptation strategy to colonize and persist in multiple niches. Thus, selection of the SCV phenotype could be exploited by P. aeruginosa to respond to various stressful conditions, including electron acceptor limitation.

However, knowledge is missing regarding the specific conditions that promote SCVs emergence or the benefit of their selection in a possible adaptive strategy. In this work, we investigated the selective pressure promoting SCV emergence in *in vitro* experiments. We chose the prototypical strain PA14, which we found readily forms SCVs in nonagitated standing culture conditions (22) to investigate the effect of O_2_ limitations on SCV emergence and survival by promoting the formation of a biofilm at the air-liquid interface.

## RESULTS

### Pellicle formation is linked to SCV incidence.

**FIG 1:**
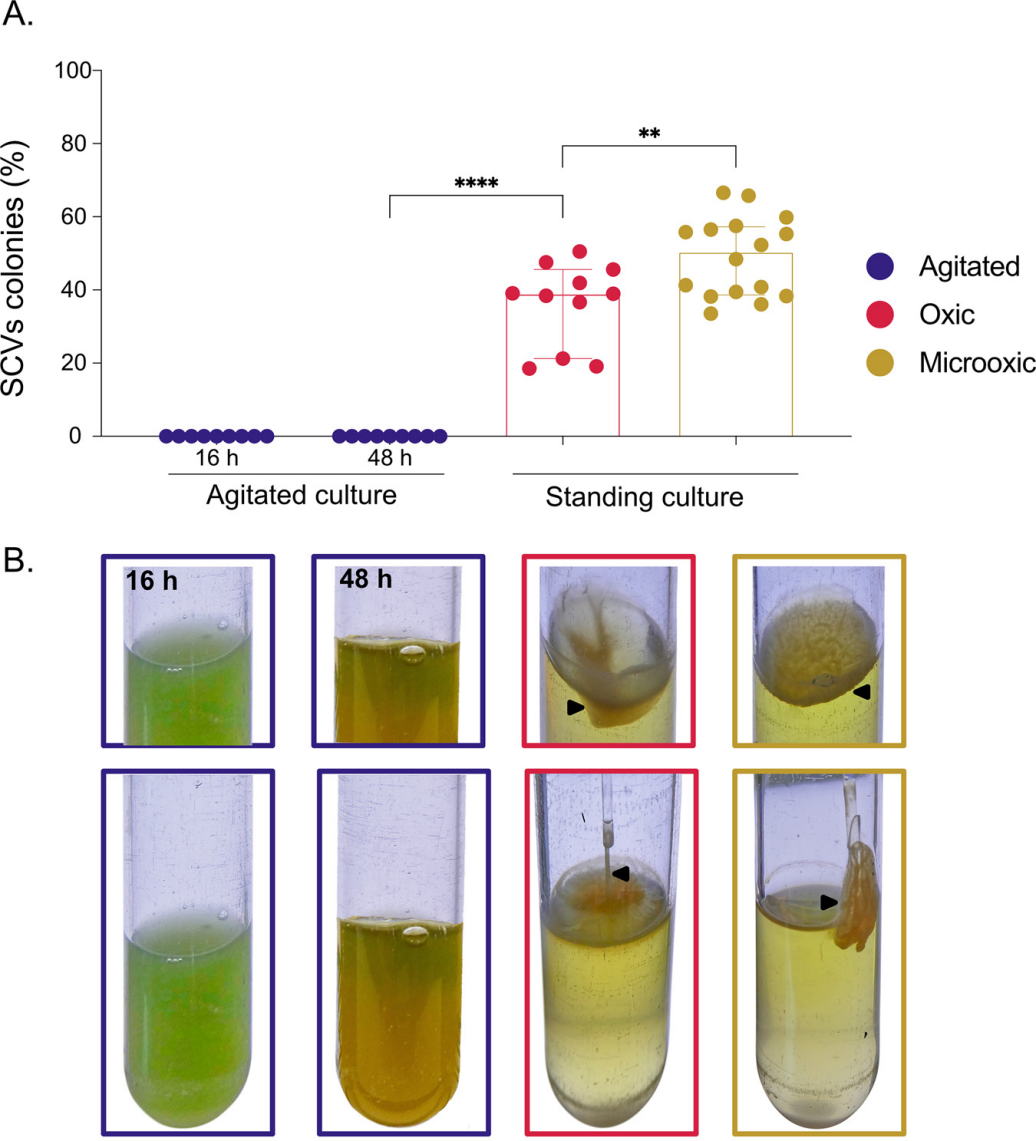
SCVs rate and growth pattern of cultures depending on O_2_ availability. P. aeruginosa PA14 was cultivated in agitated, in static oxic, or in static micro-oxic conditions in tryptic soy broth (TSB). (A) After 16 h (agitated) or 48 h (static) of incubation, ratios of small colony variant (SCV) colonies were counted. The results are expressed as percentages of the total population. Each dot represents counts for one independent culture. (B) Growth pattern of cultures incubated in agitated (purple), static oxic (pink), and static micro-oxic (yellow) conditions. A black arrowhead indicates a pellicle formed at the air-liquid surface. Pellicles were removed with a Pasteur pipette tips to gauge their cohesive properties. Note that counts and pellicles were also assessed at 48 h for the agitated culture with similar results. The statistical significance of the results was calculated by a nonparametric Mann-Whitney test. ****, *P* ≤ 0.0001; **, *P* ≤ 0.01.

SCVs of P. aeruginosa are obtained under culture conditions promoting biofilm formation (23–25). Accordingly, we do not recover SCVs from standard agitated cultures of the prototypical clinical strain P. aeruginosa PA14 even after 48 h (Fig. 1A), and there is no formation of a pellicle at the air-liquid interface (Fig. 1B). However, when PA14 is grown as a standing culture for 48 h, SCVs emerge readily and spontaneously (Fig. 1A). To confirm that colonies obtained are indeed SCVs and not merely small colonies, we performed a specific phenotypic characterization (see Text S1 and Fig. S1 in supplemental materials). The obtained colonies have a significantly smaller size than their WT counterpart does, exhibit enhanced biofilm formation, overproduce c-di-GMP, and are hyperpiliated (Fig. S1) These results confirmed that the small colonies emerging in PA14 standing cultures after 48 h are indeed SCVs. Since a surface pellicle is noticeable in standing cultures (Fig. 1B), these results indicate that SCVs emerge under conditions promoting biofilm formation.

10.1128/msphere.00057-23.1 TEXT S1 Supplementary materials and methods for Supplemental Figure S1. Download Text S1, PDF file, 0.1 MB. Copyright © 2023 Besse et al. 2023 Besse et al. https://creativecommons.org/licenses/by/4.0/ This content is distributed under the terms of the Creative Commons Attribution 4.0 International license.

10.1128/msphere.00057-23.1 FIG S1 Phenotypic features of *Pseudomonas aeruginosa* PA14 parental and small colony variant (SCV) colonies. Download FIG S1, PDF file, 0.2 MB. Copyright © 2023 Besse et al. 2023 Besse et al. https://creativecommons.org/licenses/by/4.0/ This content is distributed under the terms of the Creative Commons Attribution 4.0 International license.

Next, we asked whether biofilm-promoting conditions are exclusively responsible for the emergence of SCVs or whether an additional environmental pressure could be involved. A key difference between agitated and standing cultures is the O_2_ distribution within the liquid. Thus, we investigated the role of O_2_ in this phenomenon by cultivating P. aeruginosa PA14 under microaerobic conditions; strikingly, an even more hydrophobic and robust pellicle developed at the surface, as shown by its sticky properties when removing it with a plastic tip (Fig. 1B). Indeed, 39% of the total population of a 48-h aerobic standing culture were SCVs (Fig. 1A), while when the environmental O_2_ concentration was reduced, i.e., under microaerobic conditions, SCVs reached 50% of the total population (Fig. 1A). Since microaerobic conditions promote a higher SCV proportion (Fig. 1A) and SCVs are highly hydrophobic, this result confirms that pellicle formation is directly correlated with SCV incidence.

### Rapid depletion of oxygen in static cultures selects for SCV emergence.

**FIG 2:**
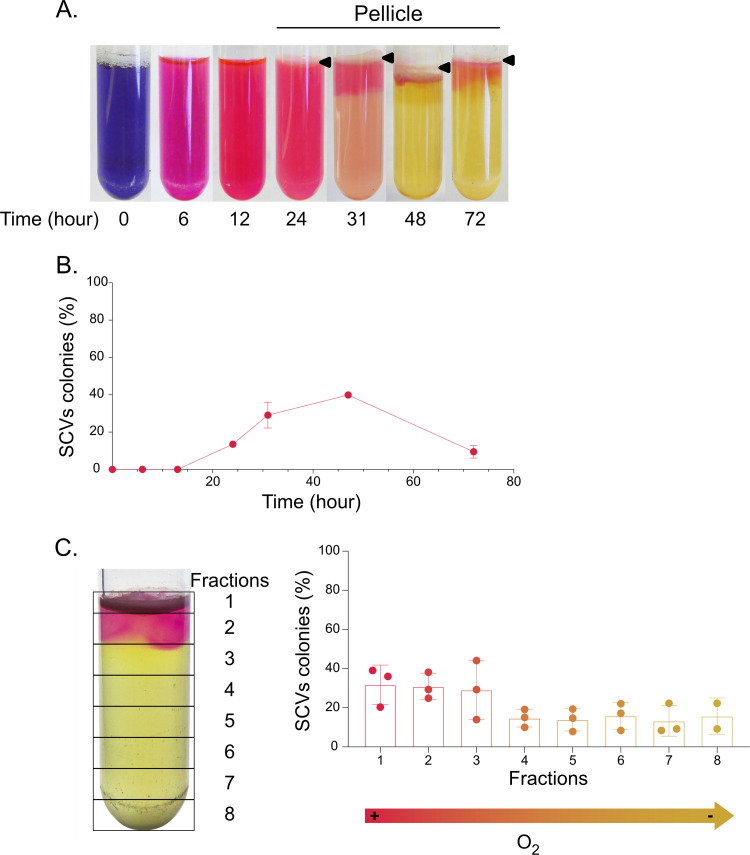
SCVs emergence and distribution in standing cultures depending on O_2_ depletion over time. PA14 was inoculated in TSB with 100 mg/mL of resazurin and incubated in aerobic static conditions. (A) After 0, 6, 12, 24, 31, 48, and 72 h, resazurin reduction was observed to assess O_2_ depletion within culture. Black arrowheads point to the pellicles formed at the air-liquid surfaces. (B) After 0, 6, 12, 24, 31, 48, and 72 h, cultures were sampled, and SCVs colonies were counted and expressed as percentages of the total population. (C) After 50 h of incubation, 1-mL fractions from the top to the bottom of the culture were sampled and spread onto tryptic soy agar (TSA) plates, and SCVs were quantified (% of total population).

To further verify whether there is a link between O_2_ availability and the incidence of SCVs in the culture, we added resazurin dye to 5-mL standing cultures. The dye turned pink as early as 6 h after inoculation, indicating that O_2_ was rapidly consumed by the bacteria (Fig. 2A). After 48 h of incubation, O_2_ was mostly depleted, as shown by the colorless (i.e., reduced) resazurin in most of the culture, and it was oxidized at the surface only (top pink layer). This indicated that O_2_ was rapidly consumed by the cells and that only surface cells could have access to significant O_2_ (Fig. 2A). Interestingly, SCVs were forming a significant portion of the whole population as soon as 24 h after the beginning of the incubation, concomitantly with the observation of a significant turbidity and prior to complete O_2_ depletion (Fig. 2A and B). The percentage of SCVs within the total population reached its maximum after 48 h of incubation, when the O_2_ was almost completely depleted (Fig. 2A and B). Since O_2_ diffusion was limited to the superficial part of the PA14 standing culture after 48 h, we sampled 1-mL fractions from the top to the bottom of the standing culture and quantified SCVs (Fig. 2C). SCVs represented ~30% of total population in the three upper fractions, in which the resazurin was still oxidized, but this percentage dropped significantly to ~15% in the deeper fractions 4 to 8 (Fig. 2C). Thus, SCVs were predominantly found at the surface, where O_2_ was still detectable and SCVs incidence was drastically reduced in parts of the culture where O_2_ was depleted (Fig. 2C).

Since SCVs promote efficient biofilm growth, we hypothesized that SCVs are functionally selected because they readily form a pellicle at the air-liquid interface, allowing rapid adaptation of the population to electron acceptor limitation by providing access that is more direct to atmospheric O_2_. Supporting this hypothesis, P. aeruginosa PA14 grows mainly as a pellicle in O_2_-limiting conditions, with almost no turbidity visible under the pellicle (Fig. 1B).

**FIG 3:**
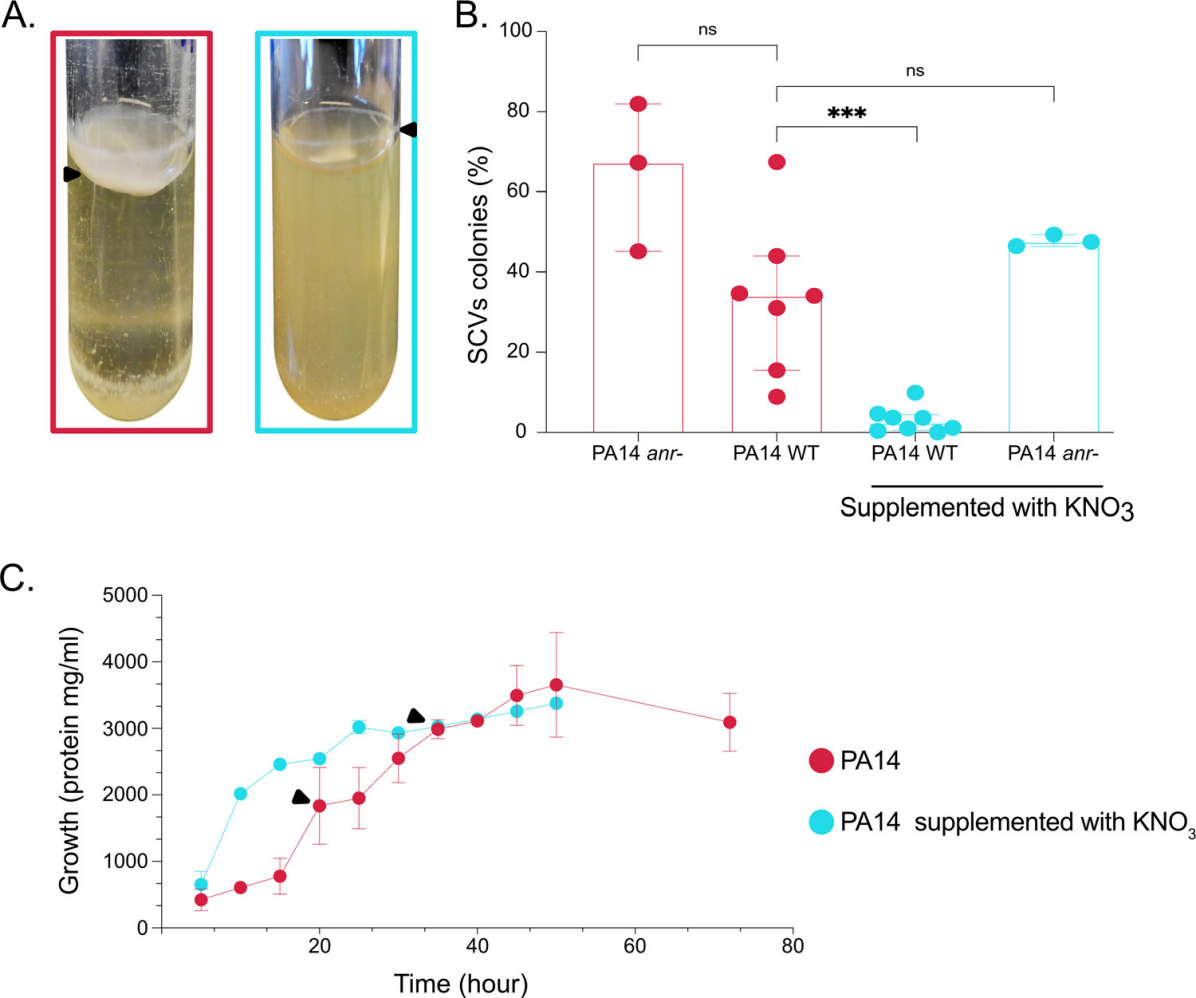
Growth pattern and SCVs emergence upon supplementation with nitrate. PA14 was inoculated in TSB or TSB supplemented with 50 mM KNO_3_. (A) Growth pattern and pellicle formation in PA14 culture not supplemented (pink) or KNO_3_ supplemented (turquoise). Black arrowheads indicate pellicle or residual pellicles formed at the air-liquid surfaces. (B) PA14 WT and PA14 *anr*− were inoculated in TSB or TSB supplemented with KNO_3_. After 48 h of incubation, the cultures were spread onto TSA plates, and the percentages of SCVs were quantified. The results are expressed as percentages of SCVs in the total population. The statistical significance of the results was calculated by a nonparametric Mann-Whitney test, ***, *P* ≤ 0.001; ns, not significant. (C) Growth of PA14 in TSB and TSB-KNO_3_ over time. Black arrowheads indicate first detection of a pellicle at the air-liquid interface. WT, wild type.

Finally, a lag phase of ~20 h was observed prior to efficient growth in standing culture (Fig. 3C). Accordingly, it is also the timing observed before the first detection of SCVs and visible occurrence of a pellicle in standing cultures (Fig. 2B), suggesting that during the lag phase observed, an initially minor SCV subpopulation is strongly positively selected to overcome survival pressure caused by O_2_ restriction. Altogether, these results indicate that restricted access to O_2_ represents a strong selective pressure to switch to the SCV phenotype in P. aeruginosa cultures.

### Presence of an homogeneously available alternative electron acceptor relieves the selective pressure to switch to SCVs.

We next wanted to determine whether the selection, caused by heterogenic O_2_ distribution in PA14 standing culture, was indeed responsible for SCV emergence and also for pellicle formation. This was verified by adding the alternative electron acceptor nitrate (as KNO_3_) to standing cultures, so that O_2_ was no longer the primary electron acceptor. Supporting our hypothesis, no clear pellicle was observed in the presence of KNO_3_, only a residual trace of a thin, fragile pellicle (Fig. 3A). This residual pellicle was nothing like the cohesive thick pellicle observed without the presence of nitrate (Fig. 3A). Also, with KNO_3_ supplementation, the culture medium was turbid, indicating that the growth was homogeneous within the culture and no longer mainly localized at the surface as observed without the addition of KNO_3_ (Fig. 3A).

Strikingly, SCVs were barely detectable in standing cultures supplemented with KNO_3_ (Fig. 3B), indicating that the presence of an homogeneously available electron acceptor prevents SCVs emergence. Anr is the main O_2_ regulator of P. aeruginosa and is activated by low O_2_ conditions to control expression of the denitrification genes, allowing the use of nitrate as electron acceptor. Thus, P. aeruginosa *anr* mutants are no longer able to use nitrate as an O_2_ alternative electron acceptor. Accordingly, the SCV level was similar between P. aeruginosa PA14 *anr*− mutant grown in KNO_3_-supplemented medium and PA14 WT grown without KNO_3_ supplementation (Fig. 3B). Altogether, these results indicate that when O_2_ limitation is overcome, the pressure to grow at the surface as a pellicle and thus switch to SCV phenotype is removed, consistent with the absence of SCVs in well agitated cultures (Fig. 1A). Limitations in an homogeneously available electron acceptor, rather than specific O_2_ limitation, is the selective pressure resulting in SCVs emergence and pellicle formation under our conditions.

### SCVs emergence is promoted in the absence of an effective electron shuttle to access O_2_.

**FIG 4:**
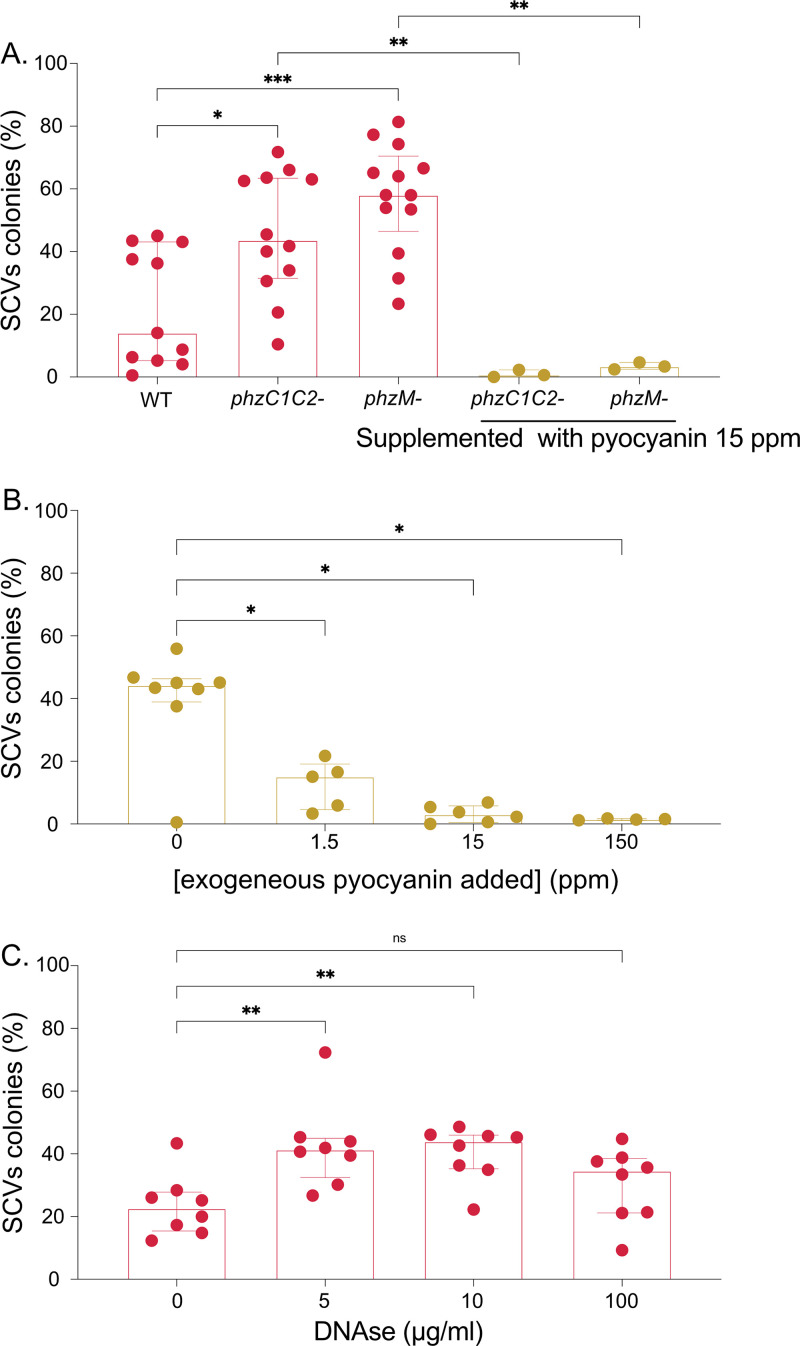
SCVs rate in cultures with an electron shuttle defect in PA14 phenazine mutants and pyocyanin or DNase-supplemented cultures. (A) PA14 WT, PA14 *phzC1C2*−, and PA14 *phzM*− were inoculated in TSB (pink) or TSB supplemented with 15 ppm of purified pyocyanin (yellow). (B) PA14 WT was inoculated in TSB supplemented with 0, 1.5, 15, or 150 ppm of purified pyocyanin. (C) PA14 WT was inoculated in TSB with 0, 5, 10, or 100 μg/mL of DNase and incubated in static conditions. After 48 h of incubation in static conditions, the cultures were spread onto TSA plates. and the percentages of SCVs colonies in the total population were quantified. Asterisks represent the statistical significance of the results calculated by a nonparametric Mann-Whitney test: ****, *P* ≤ 0.0001; ***, *P* ≤ 0.001; **, *P* ≤ 0.01; *, *P* ≤ 0.05; ns, not significant.

As previously mentioned, in biofilms, access to O_2_ is often restricted and generally localized at the surface of the growing structure. To overcome this limitation, P. aeruginosa can channel electrons from intracellular metabolism to distant extracellular oxidants through electron carriers such as phenazines (20, 33). Interestingly, growth in standing cultures of a ΔΔ*phzC1C2* mutant, unable to produce phenazines, results a higher SCVs proportions (Fig. 4A). The same result was observed for a *phzM* mutant, unable to convert phenazine-1-carboxylic acid (PCA) to pyocyanin, a blue-pigmented phenazine. Complementation of the mutation in *phz* mutants with exogenously supplemented pyocyanin inhibited the emergence for SCVs, indicating that the specific absence of pyocyanin production by these mutants promotes emergence of SCVs (Fig. 4A). Accordingly, increasing concentrations of added pyocyanin to WT standing cultures negatively correlated with the emergence of SCVs (Fig. 4B), supporting a correlation between presence of phenazines and SCVs emergence in standing liquid cultures.

To determine whether the absence of a potential efficient electron shuttle, rather than the absence of pyocyanin itself, was responsible for the higher SCVs rate, we next focused on extracellular DNA (eDNA), which enhances electron shuttle in biofilms (34). Interestingly, the addition of DNase doubled the percentage of SCVs in standing cultures (Fig. 4C), indicating that a reduction in eDNA, likely causing a reduced electron shuttle efficiency, promotes the emergence of SCVs. Altogether, these results indicate that the absence of pyocyanin, potentially impairing an efficient electron shuttle to mitigate the effects of limitations in O_2_ availability, leads to increased SCV emergence.

### Emergence of SCVs rebalances the redox state.

**FIG 5:**
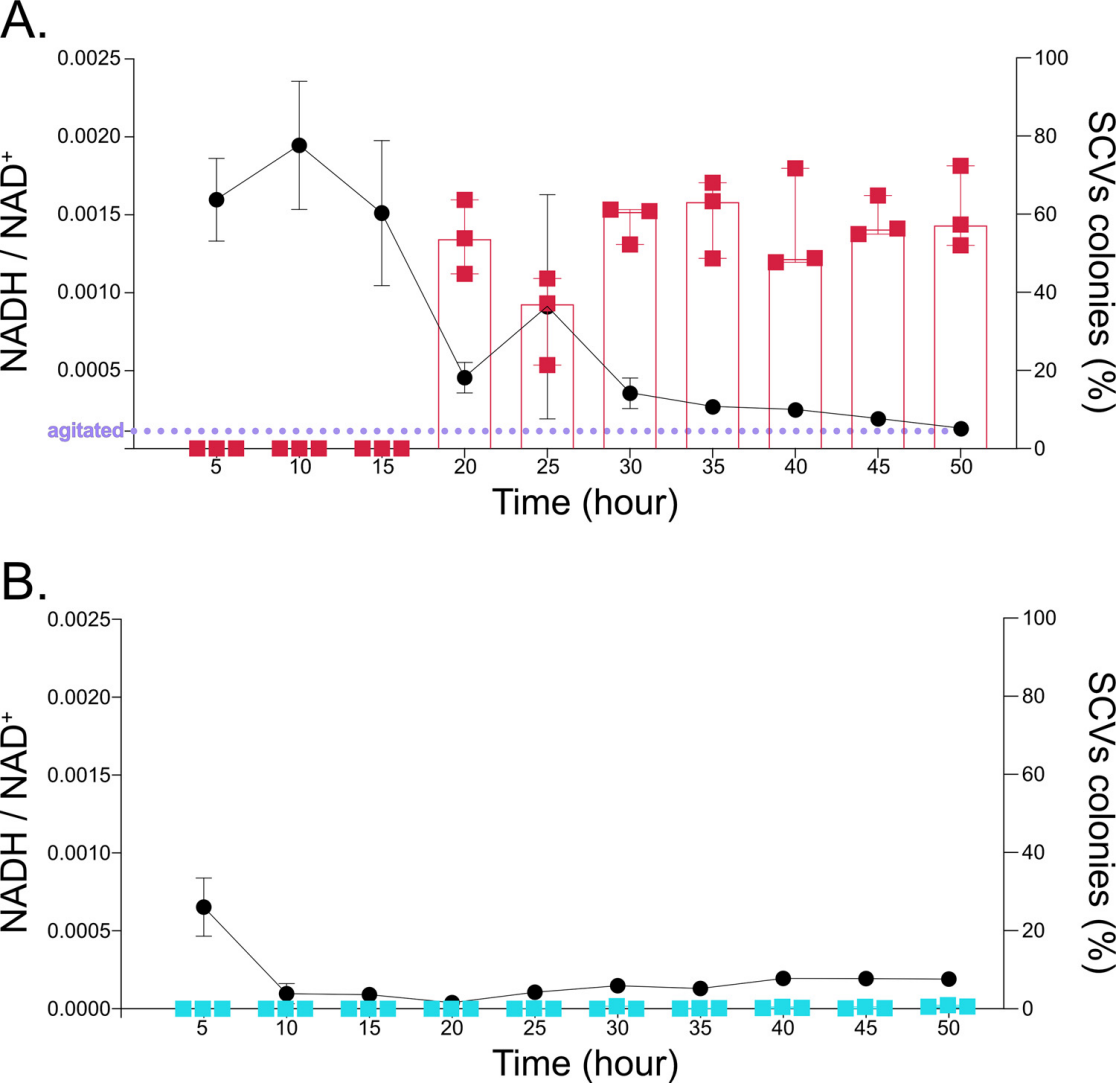
Effect of SCV emergence and KNO_3_ supplementation on the intracellular redox state of standing cultures. PA14 WT was inoculated in (A) TSB or (B) TSB supplemented with 50 mM KNO_3_. The cultures were incubated in static conditions for 50 h. The cultures were sampled at 5 h, and the percentage of SCVs colonies in the total population was determined (squares, histogram). Concomitantly, NADH and NAD^+^ were extracted from the static cultures to assess redox intracellular state (circles, curve). The purple dotted line in panel A indicates the NADH/NAD^+^ ratio of an overnight PA14 culture incubated in agitated conditions.

Restrictions in electron acceptors unbalance the intracellular redox state. Pyocyanin facilitates redox balancing, particularly in the absence of electron acceptors. Since the lack of both O_2_ or pyocyanin promotes SCVs emergence, we hypothesized that the intracellular redox state could be directly correlated with SCVs emergence. Indeed, in standing cultures in which O_2_ availability was limited, the NADH/NAD^+^ ratio was 14 times higher during the first 15 h of incubation compared with a 24-h standard agitated culture in which electron acceptors are not limiting (Fig. 5A). The same experiment was performed in tryptic soy broth (TSB) supplemented with KNO_3_. As expected, the NADH/NAD^+^ ratio was only slightly increased early on during the exponential growth phase but rapidly returned to the basal level corresponding to a 24-h standard agitated culture, confirming that the intracellular redox state is not unbalanced in conditions in which the availability of electron acceptors is not limiting (Fig. 5B). In agreement with previous observations (Fig. 1 to 3), SCVs were detected in O_2_-limiting standing cultures after 20 h of incubation (Fig. 5A), but their emergence was almost completely abrogated in static cultures supplemented with KNO_3_ (Fig. 5B). The fact that nitrate respiration also maintains a low NADH/NAD^+^ ratio indicates that it is not O_2_ limitation *per se* that is detected by P. aeruginosa and leads to selection for SCVs but rather the imbalance in the NADH/NAD^+^ ratio. Thus, selection for SCVs correlates with the intracellular oxidation state and the condition promoting the emergence of SCVs is an imbalance in intracellular redox. Since SCVs were readily detected after 20 h in the standing culture and thus emerged between the 15- and 20-h time points, we conclude that the NADH/NAD^+^ threshold sensed by P. aeruginosa WT is between 0.00151 ± 4.7E−4 and 0.00046 ± 9.8E−5 (Fig. 5). Strikingly, upon emergence of SCVs, the imbalanced NADH/NAD^+^ ratio decreased over time to finally reach the low level of the agitated culture, in nonlimiting electron acceptors conditions with a balanced ratio (Fig. 5A). This observation suggests that SCVs selection by P. aeruginosa within the population could lead to a rebalance of the intracellular state and could be exploited as an adaptive strategy by P. aeruginosa under O_2_-limited conditions.

## DISCUSSION

Energy generation in all heterotrophically growing organisms, including P. aeruginosa, is mainly based on oxidative substrate catabolism. In the respiratory cascade, O_2_ is the terminal electron acceptor (35). When O_2_ becomes limiting, intracellular accumulation of reduced compounds destabilizes the redox state, disturbing essential metabolic functions. Thus, maintaining a balanced cellular redox state is required for the survival of the cell.

In surface P. aeruginosa growing biofilms, an O_2_ gradient is forming from the top to the bottom of the dense microbial communities (20, 36). Thus, bacteria can quickly exhaust the available electron acceptors. To ensure its survival, P. aeruginosa has developed different strategies, such as pyocyanin production (17, 18) and an increase in the O_2_-exposed surface (20, 21), to mitigate the effects of low O_2_ availability and rebalance redox intracellular state in biofilms when the use of an alternative electron acceptor is impossible. However, adaptation of P. aeruginosa populations to O_2_ limitation outside the biofilm context is poorly understood. Here, we have cultivated P. aeruginosa PA14 in standing cultures, an *in vitro* mode of growth in which O_2_ is limiting. We observed spontaneous emergence of SCVs in O_2_-depleted cultures. As a direct consequence of O_2_ limitation, the redox intracellular state was imbalanced in standing cultures in the absence of an alternative electron acceptor. We have demonstrated that in an O_2_-limited environment, P. aeruginosa PA14 senses an increase in the NADH/NAD^+^ ratio to select SCVs. This redox threshold was not reached in the presence of a functional Anr regulator, allowing the use of nitrate as an alternative electron acceptor and resulting in the abolishment of SCV emergence. Thus, nitrate respiration prevents SCV formation presumably by maintaining low NADH levels. In addition to Anr directly sensing O_2_ concentration, other regulators are involved in P. aeruginosa redox balancing. For instance, the two-component system RoxSR is likely to sense redox status in P. aeruginosa and consequently regulate the expression of terminal oxidases, as well as other genes involved in the respiratory function such as *hemB* and *nuoAL* (37). Also, the SCV emergence rate was directly linked to the strength of the survival pressure induced by the intracellular redox imbalance, as indicated by the increase in SCV proportion under microaerobic conditions or in the absence of phenazines.

SCV emergence was correlated with a rebalance of the intracellular redox state and formation of a pellicle at the air-liquid interface. Since SCVs display mutations in *Yfi* or *Wsp* systems (22), leading to the production of EPS (38), i.e., *pel* in P. aeruginosa PA14 (39), one can easily conclude that SCV emergence allowed bacterial population survival by promoting the formation of a pellicle at the air-liquid interface to access the electron acceptor and thus rebalance the redox state. Is supporting a mode of growth to access O_2_ when the redox state is imbalanced the sole benefit of SCV selection? Or do SCVs also overexpress functions involved in respiration process regulation, such as the known ones Anr, RoxSR, RpoS, or Fur? Transcriptomic analyses did not reveal such changes in gene expression (40, 41). However, after 72 h of static incubation, the percentage of SCVs in the total population dropped, correlating with the beginning of the stationary phase. Thus, we hypothesize that in the stationary phase, the SCV phenotype does not provide as much benefit to the whole population and an alternative adaptive mechanism takes over to extend population survival. This is the first study reporting a direct correlation between SCV emergence and O_2_ limitation/redox imbalance and to point out the involvement of SCVs in an adaptive response to maintain a balanced redox state.

Emergence of an alternative distinguishable morphotype from WT, called Wrinkly Spreader (WS), under very similar O_2_ limited standing liquid conditions has been previously reported for the species Pseudomonas fluorescens (42). As for P. aeruginosa SCVs, the emergence of WS is specifically driven by O_2_ depletion gradients in P. fluorescens cultures (43). WS emergence was also favored near the air-liquid interface, as is the case for SCVs. The authors of those studies have demonstrated that initial colonizing bacteria within static cultures established the O_2_ gradient before significant growth had occurred. The high O_2_ niches near the surface favored the emergence of the WS phenotype (43). A very similar scenario occurs in our standing cultures, in which SCVs emerge mainly in the O_2_-enriched upper fractions within an O_2_ heterogeneous culture. While arising under similar conditions, WS and SCV are very distinct phenotypes since, nonexhaustively: (i) the WS phenotype does not necessarily exhibit a small colony (44), while it is the primary feature of the SCV phenotype; (ii) despite the fact that WS and SCV result from alteration of c-di-GMP regulation systems, in WS, this leads to overproduction of cellulose responsible for wrinkles, when the mechanism is quite different in SCVs of P. aeruginosa with cellulose not being involved in this phenotype (45); and (iii) the presence of wrinkles at the colony surface is not a specific feature associated with a single phenotype, since a wrinkly phenotype arises frequently during evolution of P. aeruginosa (46). Thus, emergence of either SCV or WS is likely to benefit the whole population in standing cultures of P. aeruginosa and P. fluorescens, respectively, but the exact conditions promoting their emergence (i.e., redox imbalance for P. aeruginosa, while to date only O_2_ limitation is reported for P. fluorescens) and the adaptive response mechanism could be different. Still, the fact that both morphotypes result from overproduction of c-di-GMP suggests a form of convergent evolution.

In addition to O_2_ limitation leading to redox imbalance, the SCV phenotype can also be the result of exposures to different stresses. In one of the first report of SCV phenotype, Déziel et al. (23) obtained SCVs by growing P. aeruginosa on an extremely hydrophobic source of carbon, hexadecane. In addition, accumulation of the toxic intermediate, gluconolactone, following knockout of the gluconolactonase coding gene *ppgL*, resulted in SCV emergence (47). Exposure to a sublethal hydrogen peroxide level (H_2_O_2_) concentration during 120 generations also led to the emergence of SCVs (48). In all these scenarios, forming a biofilm is the best solution to ensure survival. For instance, to grow on hexadecane, the only way to thrive was to grow directly attached to the substrate; thus, there is a need for rapid biofilm formation. This is similar to our O_2_-depleted standing cultures, in which growth as a biofilm at the air-liquid interface allows for increased access to O_2_. Thus, SCVs emergence could rather not only be a way to promote biofilm formation but instead an essential process to readily form a biofilm and ensure survival or a way to adapt to various stress conditions.

In P. aeruginosa natural habitats, SCV emergence could play a key role in an adaptive strategy. One of the most studied P. aeruginosa habitats is CF-infected hosts. P. aeruginosa is a key pathogen in people with CF. Upon infection, P. aeruginosa colonizes CF mucus, a specific microenvironment displaying O_2_ gradient with O_2_-limited environments to anaerobic environments (49). P. aeruginosa can grow in the hypoxic/anaerobic CF mucus. Anaerobic growth may be in part supported by the terminal electron acceptor nitrate, allowing denitrification to generate energy (49, 50). Interestingly, SCVs have been reported several times from CF samples (25, 51–53) and could also be part of the redox stress response to hypoxia, largely responsible for the formation of biofilm, the predominant phenotype of P. aeruginosa in CF airways (54). Thus, we speculate that the stress of hypoxic/anaerobic environments induces P. aeruginosa to acquire phenotypic features, such as the switch to the SCV phenotype, that allow it to enhance biofilm formation and evade hosts defenses and antimicrobial treatments, ensuring its survival. A correlation between the emergence of P. aeruginosa SCVs and infection persistence in animal models was established, supporting the idea that the SCV phenotype confers a fitness advantage under chronic infection conditions (55–57). Since P. aeruginosa is the main cause of morbidity and mortality in CF individuals, and infections are becoming more difficult to treat because of the resistance to many antibiotics (58, 59), a better understanding of the pathways that contribute to survival and virulence of P. aeruginosa during infection, e.g., mechanisms of adaptation in O_2_-starved environments, is central to developing strategies to combat human P. aeruginosa infection.

In conclusion, our data show that P. aeruginosa PA14 SCVs emerge in response to an imbalanced redox state resulting from limitations in electron acceptor availability and that their emergence is correlated with formation of a surface pellicle, i.e., a biofilm-like structure, at the air-liquid interface. This allows access to O_2_ and a rebalance of the intracellular state, ensuring survival of P. aeruginosa. Here, we pointed out a specific stressing condition leading to SCV emergence, but other stresses could result in SCV emergence. We propose that SCVs emergence is likely to be central in the adaptive response of P. aeruginosa, accounting for its versatility and persistence in a variety of environments.

## MATERIALS AND METHODS

### Bacterial strains and liquid growth conditions.

The Bacterial strains are listed in Table 1. “Agitated culture” refers to strains grown in 5 mL TSB (BD) at 37°C in a TC-7 roller drum (New Brunswick) at 240 rpm for 16 h. “Standing culture” refers to strains grown in static conditions at 34°C for up to 96 h. Standing cultures were grown in 5 or 8 mL TSB. When needed, TSB was supplemented with 50 mM KNO_3_, purified pyocyanin (chloroform extraction from a PA14 culture) or DNase (Sigma). Standing cultures were inoculated at an initial optical density at 600 nm (OD_600_) of 0.05 and placed in an Infors incubator (Multitron Pro) without agitation, corresponding to “oxic conditions,” or in an anaerobic jar with a lighted candle to consume ambient O_2_, corresponding to “micro-oxic conditions.”

**TABLE 1:** Bacterial strains used in this study

P. aeruginosa strain	Relevant characteristics	Lab collection strain no.	Reference
PA14 wild type	Clinical isolate UCBPP-PA14	ED14	(11)
PA14 *anr*::MrT7	MrT7 transposition insertion mutant; Gm^r^ mutant ID 26855	ED1092	(61)
PA14 ΔΔ*phzC1C2*	Double deletion mutant of *phzC1* and *phzC2*	ED85	Unpublished lab strain
PA14 *phzM*::Gm	Gentamicin-resistant cassette inserted into *phzM*; Gm^r^	ED120	Gift from Pierre Cornelis

### Isolation of SCVs.

Selection of SCVs in standing cultures was performed following the method we described previously (22). Briefly, overnight (O/N) cultures of parental strain were grown at 30°C in an Infors incubator (Multitron Pro) at 180 rpm in angled tubes. O/N cultures were used to inoculate fresh TSB at an initial OD_600_ of 0.05. As previously described, the tubes were incubated without agitation at 34°C for up to 96 h. Standing cultures were then sampled to perform phenotypic tests, quantify SCVs or quantify total proteins (Bradford method) for growth assessment.

### Visualization and quantification of SCVs.

To increase reproducibility of the results, the cultures were sampled at ~5 mm under the surface (underneath the pellicle), unless otherwise stated. A sterile P1000 tip with a cut end was added in the culture tube prior to inoculation to facilitate sampling through the tip and avoiding collection of the sticky pellicle. About 100 to 150 μL of the culture were sampled with a sterile Pasteur pipette and thoroughly mixed by pipetting up and down to homogenize sample and get rid of potential aggregates. Then, 20 μL were collected from the homogenized sample and diluted in TSB. Finally, 50 μL of the diluted sample were spread on tryptic soy agar (TSA) plates solidified with 2% agar (AlphaBiosciences). The plates were incubated for 24 h at 30°C. CFU (total + SCVs) colonies were counted manually from photographs using ImageJ. The results are expressed in SCVs percentage of total population. All experiments were performed at least in triplicate.

### Detection of O_2_ availability.

To assess O_2_ consumption, we used the O_2_-sensitive dye resazurin. In the presence of O_2_, blue resazurin is irreversibly reduced to pink-colored resorufin, which is further reversibly reduced to colorless dihydroresorufin under reducing conditions, such as when O_2_ is depleted. TSB was supplemented with 100 mg/mL of sterile resazurin prior to inoculation of standing cultures. Resazurin reduction was assessed visually.

### Bradford protein assay.

Due to the highly aggregative properties of SCVs, OD_600_ measurements were not appropriate to evaluate the growth of standing cultures. The Bradford protein assay was used to quantify the total proteins concentration in all our samples. Pellets from 1 mL of culture were resuspended in 1 mL of 0.1 N NaOH and incubated 1 h at 70°C, and then protein concentrations were measured in samples according to the manufacturer guidelines for the Bradford reagent (Alfa Aesar).

### Extraction and quantification of intracellular of NADH and NAD^+^.

Extraction of NADH and NAD^+^ was carried out according to the method described by Kern et al. (60). Briefly, 5-mL standing cultures were vortexed. Two 1.8-mL samples of culture were centrifuged 1 min at 16,000 × *g*. The pellets were resuspended in 200 μL of 0.2 M NaOH (for extraction of NADH) or 200 μL of 0.2 M HCl (for extraction of NAD^+^). The extracts were incubated 10 min at 50°C, followed by 5 min on ice. To neutralize solutions, 200 μL of 0.1 M HCl or 200 μL of 0.1 M NaOH were added one droplet at the time while vortexing. The samples were centrifuged for 5 min at 16,000 × *g*, and the resulting supernatants were collected and stored at −80°C until quantification.

For quantification, each step was performed protected from light. A total of 80 μL of a master reagent mix (20 μL of 1 M bicine buffer [pH 8, Sigma-Aldrich], 10 μL H_2_O, 10 μL of 40 mM EDTA, 10 μL of 100% ethanol, 10 μL of 4.2 mM thiazolyl blue tetrazolium bromide (Sigma-Aldrich), and 20 μL of 16.6 mM phenazine ethosulfate [Sigma-Aldrich]) was dispensed into individual wells of a 96-well plate. Then 15 μL of samples or standards (pure NADH and NAD^+^, Sigma-Aldrich) were added to each well. Each plate was incubated 10 min at 30°C. The reaction was started by the addition of 5 μL alcohol dehydrogenase (Sigma-Aldrich A-3263) (1 mg/mL in 0.1 M bicine [pH 8]). The absorbance was measured at 570 nm every 30 s for 45 min with a microplate reader (Cytation3, Biotek). The slopes were generated from standards and samples absorbance measures at 570 nm over time. Standard curves were generated from NADH and NAD^+^ standard slopes and used to calculate absolute concentrations of NADH and NAD^+^ in samples. The values were normalized to the protein concentration of the original cell culture sample and correlated with the percentage of SCVs in the same cell culture.
