# Supplementary material for: Emergence of Small Colony Variants Is an Adaptive Strategy Used by Pseudomonas aeruginosa to Mitigate the Effects of Redox Imbalance
Source: mSphere. 2023 Feb 28;8(2):e00057-23. doi: 10.1128/msphere.00057-23 (PMC10117050; doi:10.1128/msphere.00057-23)
Supplement: TEXT S1 [file msphere.00057-23-s0001.docx]

SUPPLEMENTARY MATERIALS AND METHODS

Isolation of SCVs. Static cultures of PA14 (parental strain, *Pseudomonas aeruginosa* UCBPP-PA14 (1) were inoculated at an initial OD_{600} of 0.05 and incubated at 34°C for 50 h. Cultures were then spread on tryptic soy 2% agar plates (TS-Agar; AlphaBiosciences), unless stated otherwise. Two percent agar was utilized to limit expansion of colonies and improve isolation of the distinct morphotypes. Colony diameters were measured using ImageJ after incubation for 48 h at 30°C.

Phenotypic tests. Phenotypic tests were performed on small colonies to confirm they are SCVs. Since the SCV morphotype was easily recognizable among others (dark small colonies with sharp edges) and *P. aeruginosa* PA14 does only produce SCVs as small colonies in these standing conditions (2), the phenotypic tests were performed only for the first experiment. PA14 parental strain and its isolated small colonies were grown in TSB at 30°C, in an Infors incubator (Multitron Pro) at 180 rpm in angled tubes, overnight (O/N). Cultures were then transferred to clean tubes to perform experiments or Bradford protein quantifications. Each phenotypic test was performed in technical triplicates and the statistical significance of the results were calculated using Ordinary one-way analysis of variance (ANOVA).

Swimming motility tests. Swimming was performed as previously described (2). Briefly, 2.5 µL of culture was inoculated in agar of swim plates (20 mM NH_4Cl, 12 mM Na_2HPO_4, 22 mM KH_2PO_4, 8.6 mM NaCl, 0.5% Casamino acids (CAA), 0.3% Bacto-Agar (BD), supplemented with 1 mM $MgSO_4$, 1 mM $CaCl_2$ and 11 mM dextrose), previously dried for 15 min under the flow of a biosafety cabinet. After 20 hours of incubation at 30°C, the area of the turbid circular zone was measured (mm^2) using ImageJ. All experiments were performed in triplicates.

Biofilm formation. Biofilm were grown in microtiter (96-well) plates containing 1/10 TSB supplemented with 0.5% CAA and inoculated with O/N cultures of parental or small colonies PA14 at a concentration of 70 mM proteins. Each sample was inoculated in five different wells. After 24 h of incubation at 30°C without agitation, plates were rinsed thoroughly with distilled water. 200 µL of a 1% crystal violet solution was added to each well and plates were incubated at room temperature for 15 minutes. Plates were then rinsed thoroughly with distilled water and the dye was solubilized in 300 µL in 30% acetic acid. The absorbance was measured at 590 nm with a microplate reader (Cytation3, Biotek).

Pyoverdine production. Pyoverdine production was measured as previously described (2). Briefly, 200 µL of culture was transferred in black 96-well plates (Greiner). Fluorescence was measured at excitation/emission wavelengths of 390 nm/530 nm using a multimode microplate reader (Cytation3, Biotek).

C-di-GMP quantification. Intracellular levels of c-di-GMP were assessed as described (2). Briefly, PA14 parental strain and 3 small colonies obtained from static cultures were transformed with purified pCdrA-gfpC (3,4) by electroporation. Three of the transformation clones grown on TS-Agar 2% supplemented with 100 µg/ml gentamycin, were selected and cultured in TSB supplemented with gentamycin 100 µg/ml. Cultures were washed twice in fresh TSB before fluorescence was measured in black 96-well plates (Greiner) at excitation/emission wavelengths of 490 nm/515 nm using a Cytation3 microplate reader (BioTek). Fluorescence from non-transformed strain was subtracted from the fluorescence signal for the transformed strains.

Transmission Electron Microscopy. SCVs and parental colony were inoculated in 5 mL of fresh TSB and incubated at 30°C in a TC-7 roller drum (New Brunswick) at 240 rpm for 9 h. Cultures were then homogenized by very gently hand-mixing to avoid pili breaking. Homogenized cultures were fixed with phosphotungstic acid for 5 minutes and placed on 200 mesh copper grids. Samples were observed with a high voltage of 75 kV.

REFERENCES FOR SUPPLEMENTARY MATERIALS AND METHODS

1. Rahme LG, Stevens EJ, Wolfort SF, Shao J, Tompkins RG, Ausubel FM. 1995. Common virulence factors for bacterial pathogenicity in plants and animals. Science 268:1899-902.
2. Besse A, Groleau MC, Trottier M, Vincent AT, Déziel E. 2022. *Pseudomonas aeruginosa* strains from both clinical and environmental origins readily adopt a stable small-colony-variant phenotype resulting from single mutations in c-di-GMP pathways. J Bacteriol 204:e0018522.
3. Rybtke M, Chua SL, Yam JKH, Givskov M, Yang L, Tolker-Nielsen T. 2017. Gauging and Visualizing c-di-GMP Levels in *Pseudomonas aeruginosa* Using Fluorescence-Based Biosensors. Methods Mol Biol 1657:87-98.
4. Rybtke MT, Borlee BR, Murakami K, Irie Y, Hentzer M, Nielsen TE, Givskov M, Parsek MR, Tolker-Nielsen T. 2012. Fluorescence-based reporter for gauging cyclic di-GMP levels in *Pseudomonas aeruginosa*. Appl Environ Microbiol 78:5060-9.
