# Supplementary material for: Emergence of Small Colony Variants Is an Adaptive Strategy Used by Pseudomonas aeruginosa to Mitigate the Effects of Redox Imbalance
Source: mSphere. 2023 Feb 28;8(2):e00057-23. doi: 10.1128/msphere.00057-23 (PMC10117050; doi:10.1128/msphere.00057-23)
Supplement: FIG S1 [file msphere.00057-23-s0002.pdf]

SUPPLEMENTAL FIGURE

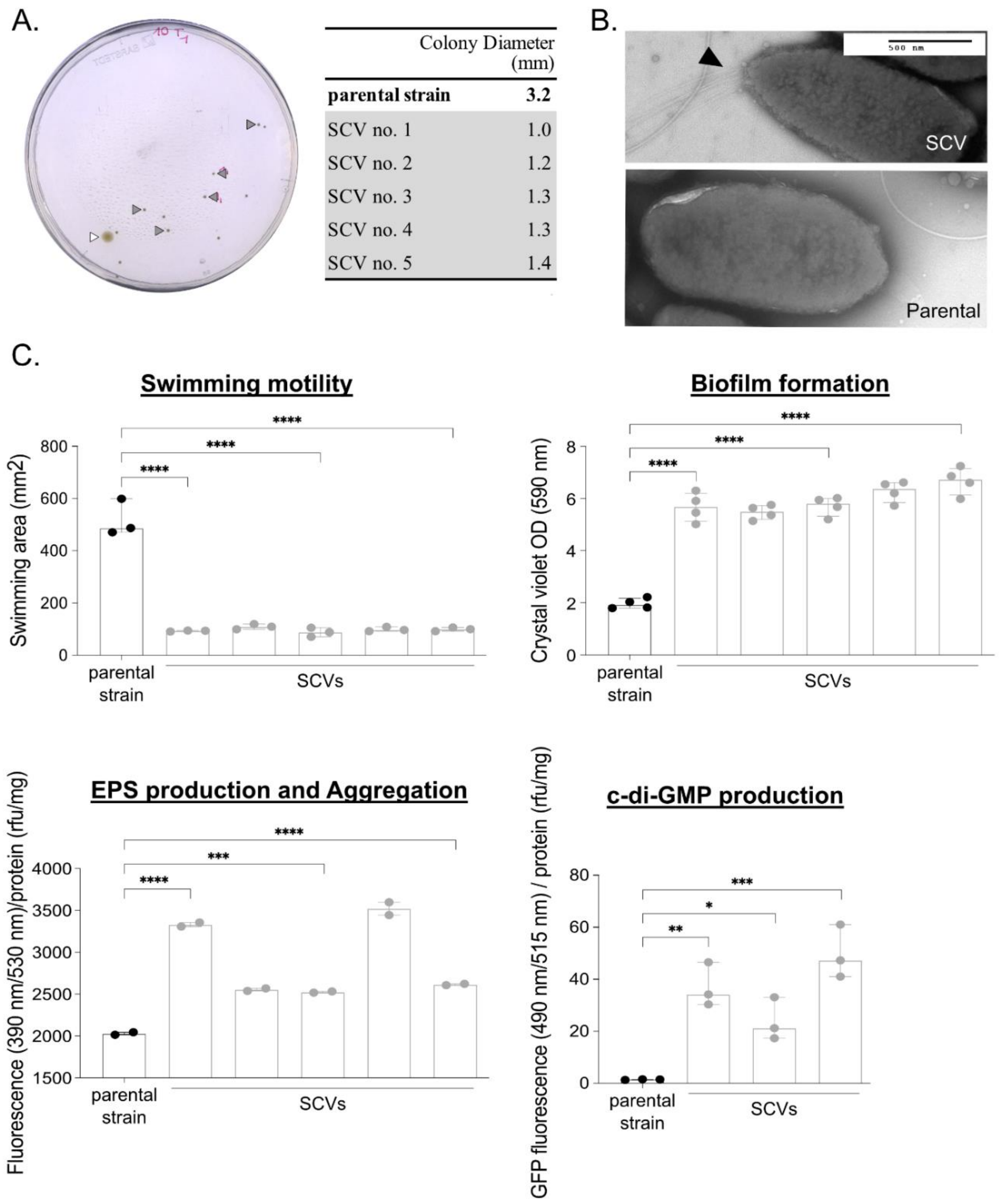

	Colony Diameter (mm)
**parental strain**	**3.2**
SCV no. 1	1.0
SCV no. 2	1.2
SCV no. 3	1.3
SCV no. 4	1.3
SCV no. 5	1.4

Figure S1. Phenotypes features of PA14 parental and SCV colonies. Static cultures of PA14 were plated after 50 h of incubation. Colonies obtained (parental and SCVs isolates) were measured (A) and collected to perform phenotypic tests (C). Stars represents the statistical significance of the results calculated by an Ordinary one-way analysis of variance (ANOVA),

****, P Value ≤ 0.0001; ***, P Value ≤ 0.001; **, P Value ≤ 0.01, *, P Value ≤ 0.1. SCVs and parental strains were visualized by TEM (B). Black arrow indicates presence of pili.
